# Retraction of “Hydrogenolysis of Polyethylene and Polypropylene into Propane over Cobalt-Based Catalysts”

**DOI:** 10.1021/jacsau.4c00090

**Published:** 2024-02-07

**Authors:** Guido Zichittella, Amani M. Ebrahim, Jie Zhu, Anna E. Brenner, Griffin Drake, Gregg T. Beckham, Simon R. Bare, Julie E. Rorrer, Yuriy Román-Leshkov

The authors retract this Letter (DOI: 10.1021/jacsau.2c00402) due to newly uncovered data that invalidate the original claims of the published article where high propane selectivity values have been reported. New analytical protocols reveal the actual product distribution to be predominantly C_3_–C_7_ species. The following analytical errors led to propane selectivity overestimation:

(i) **Product Identification** The original analysis involved the deconvolution of a GC-FID signal of C_1_–C_3_ species using GC-TCD signals. The authors have since adopted a GC-FID configuration capable of separating all light hydrocarbon species and found that the isobutane signal (not detected by the GC-TCD method used in the initial publication) was overlapping with the “C_1_–C_3_” signal. Hence, the overlapping *n*-butane was also erroneously included in the “C_1_–C_3_” signal, attributing C_4_ yields primarily to propane.
(ii) **Headspace Sampling** The original headspace capture method sampled a portion of the headspace from the pressurized reactor with a gas bag and discarded the remainder, assuming a consistent composition. The authors have found this method produces a gas sample richer in propane and a remainder richer in butanes. This may be due to the difference in the vapor–liquid equilibrium of the light hydrocarbons as the high-pressure reactor is vented into a gas bag. Complete capture of the reactor headspace avoids this issue.
(iii) **Workup Losses** The reported mass balances of the original work were sometimes low and it was assumed the headspace composition was correct but that the total headspace mass was reduced due to workup losses. The authors have since found that lost mass is likely associated with moderately volatile species (C_5_–C_7_) not effectively captured in headspace or liquid workup. The precise identification of these missing species remains challenging. Since selectivity was computed on a products-recovered basis, poor mass balances favored propane selectivity.

The original Letter reported the inclusion of Co for improving selectivity. With newly developed procedures, the authors have determined that experiments with Co-containing catalysts exhibit lower mass balances, likely due to workup losses, which in combination with product identification errors, previously generated artificially high propane selectivity values. Instead, the addition of Co tends to shift overall selectivity to larger hydrocarbon products.

The original Letter was published on October 5, 2022, and retracted on February 7, 2024.

